# Global prevalence and trend of anxiety among graduate students: A systematic review and meta‐analysis

**DOI:** 10.1002/brb3.2909

**Published:** 2023-02-27

**Authors:** Ting Chi, Luying Cheng, Zhijie Zhang

**Affiliations:** ^1^ Comprehensive Rehabilitation Center, Gansu Provincial Cancer Hospital Gansu China; ^2^ School of Nursing Lanzhou University Lanzhou Gansu China; ^3^ Evidence Based Medicine Centre Lanzhou University Lanzhou Gansu China; ^4^ Department of Sino‐French Neurological Rehabilitation, Gansu Provincial People's Hospital Gansu China

**Keywords:** anxiety disorder, graduate students, mental health, meta‐analysis, systematic review

## Abstract

**Objective:**

To evaluate the comprehensive prevalence of anxiety among postgraduates and estimate its changes with a meta‐analysis.

**Method:**

Systematic retrieval to SAGE, ERIC, EBSCO, Wiley, ScienceDirect, ProQuest, PubMed, EMBASE, and Web of Science database was performed for quantitative studies on the prevalence of anxiety among graduate students published before November 22, 2022. The prevalence of anxiety synthesized with random‐effects model, and subgroup analysis was conducted by study characteristics (publication year, sampling method, and measurements) and subjects’ characteristics (gender, region, and educational level).

**Result:**

Fifty studies were included in the meta‐analysis, totaling 39,668 graduate students. The result revealed that 34.8% of graduates suffered from the anxiety (95% CI: 29.5%–40.5%). Specifically, 19.1% (95% CI: 15.4%–23.5%) had mild anxiety, 15.1% (95% C: 11.6%–19.6%) had moderate anxiety, and 10.3% (95% CI: 7.2%–14.6%) had severe anxiety. And this prevalence showed a upward trend since 2005. Besides, master students suffered slightly less than doctoral students (29.2% vs. 34.3%), and female had similar anxiety to male (26.4% vs. 24.9%). Due to the COVID‐19, the prevalence of anxiety is higher after the pandemic than that before (any anxiety: 34.3% vs. 24.8%). Compared with other countries, students from Saudi Arabia, India, and Nepal were more vulnerable. The results of quality assessment showed that, 5 (10%) were in high quality, 21 (42%) were in moderate to high quality, 21 (42%) were in low to moderate quality, and 3 (6%) were in low quality. But, the studies with low quality tend to report a higher prevalence than that with high quality (40.3% vs. 13.0%), studies with nonrandom sampling tend to report a higher prevalence than that with random sampling (33.6% vs. 20.7%). Although we included the data collected based on the standard scales, there were higher heterogeneity among the measure (*Q* = 253.1, *df* = 12, *p* < .00).

**Conclusion:**

More than one‐third postgraduates suffered from anxiety disorder, and this prevalence had a slight upward trend since 2005, school administrators, teachers and students should take joint actions to prevent mental disorder of graduates for deteriorating.

## Graphical abstract

Anxiety is the early manifestation of most psychological disorders, such as depression and obsessive‐compulsive disorder. Chronic environmental stress is main factor lead to anxiety, while stress among academics is alarmingly widespread, especially in postgraduate students, a group that typically faces high levels of job insecurity and unbalance between life and work. This study aimed toevaluate the comprehensive prevalence of anxiety among postgraduates and estimate its changes with a meta‐analysis.

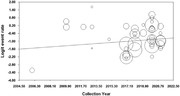

## 1 INTRODUCTION

Anxiety, characterized as the continuous feelings of nervousness, trembling, together with fears, worries, and forebodings (Testa et al., 2013). In addition to intense feelings of fear or panic, people who suffered anxiety distress may experience other physical symptoms, such as fatigue, dizziness, headache, nausea, abdominal pain, palpitations, shortness of breath, and urinary incontinence (Eysenck et al., 2007; Moran, 2016). Anxiety is the early manifestation of most psychological disorders, such as depression and obsessive‐compulsive disorder (Marty & Segal, 2015). At worst, anxiety can even lead to suicide. While it took little attention in the general population, and often undetected and severely under‐treated (Kroenke et al., 2007). As WHO's report, 264 million of people living with anxiety disorders in the world, increased by 14.9% since 2005, ranked as the sixth‐largest contributor to nonfatal health loss globally and appear in the top 10 causes of Years Lived with Disability (YLD), in all WHO regions (WHO, 2017).

Chronic environmental stress is main factor lead to anxiety, while stress among academics is alarmingly widespread (Bozeman & Gaughan, 2011; Reevy & Deason, 2014), especially in postgraduate students (Kinman, 2001), a group that typically faces high levels of job insecurity and unbalance between life and work. As reported that, 85% of postgraduate students spent 41+ h a week on their postgraduate program, still, 74% of them were inability to finish studies in the set time, and 79% had uncertainty about their job and career prospects (Woolston, 2019), which evoked widely concern on the anxiety disorder among them.

Surprising but is expected, the anxiety in graduate students was six times than the general population (41% vs. 6%) (Evans et al., 2017). Numerous studies also explored the prevalence of anxiety disorder among them, while the observed prevalence of anxiety varied from 9.2% (Wang et al., 2018) to 86.0% (Garcia‐Williams et al., 2014), and the trajectory of the change remained unclear. A reliable estimation of anxiety prevalence of graduate students and its changes is essential to inform tailored efforts to prevent, identify, and treat mental distress, and to design a suitable public health policy.

## 2 OBJECTIVES

A comprehensive estimate of the anxiety prevalence and the change through the years is essential to inform targeted efforts to prevent and identify anxiety among graduate students. The present study systematically reviewed the publications on the anxiety disorder among graduate students to answer the following questions:

1. What is the prevalence of anxiety for graduate students?
2. How has the prevalence of anxiety changed through the year?
3. Is the prevalence of anxiety affected by the measure or sampling methods?
4. Is the prevalence of anxiety moderated by gender, education level, or region?

## 3 METHODS

This study was not preregistered.

### 3.1 Literature retrieve

**FIGURE 1:**
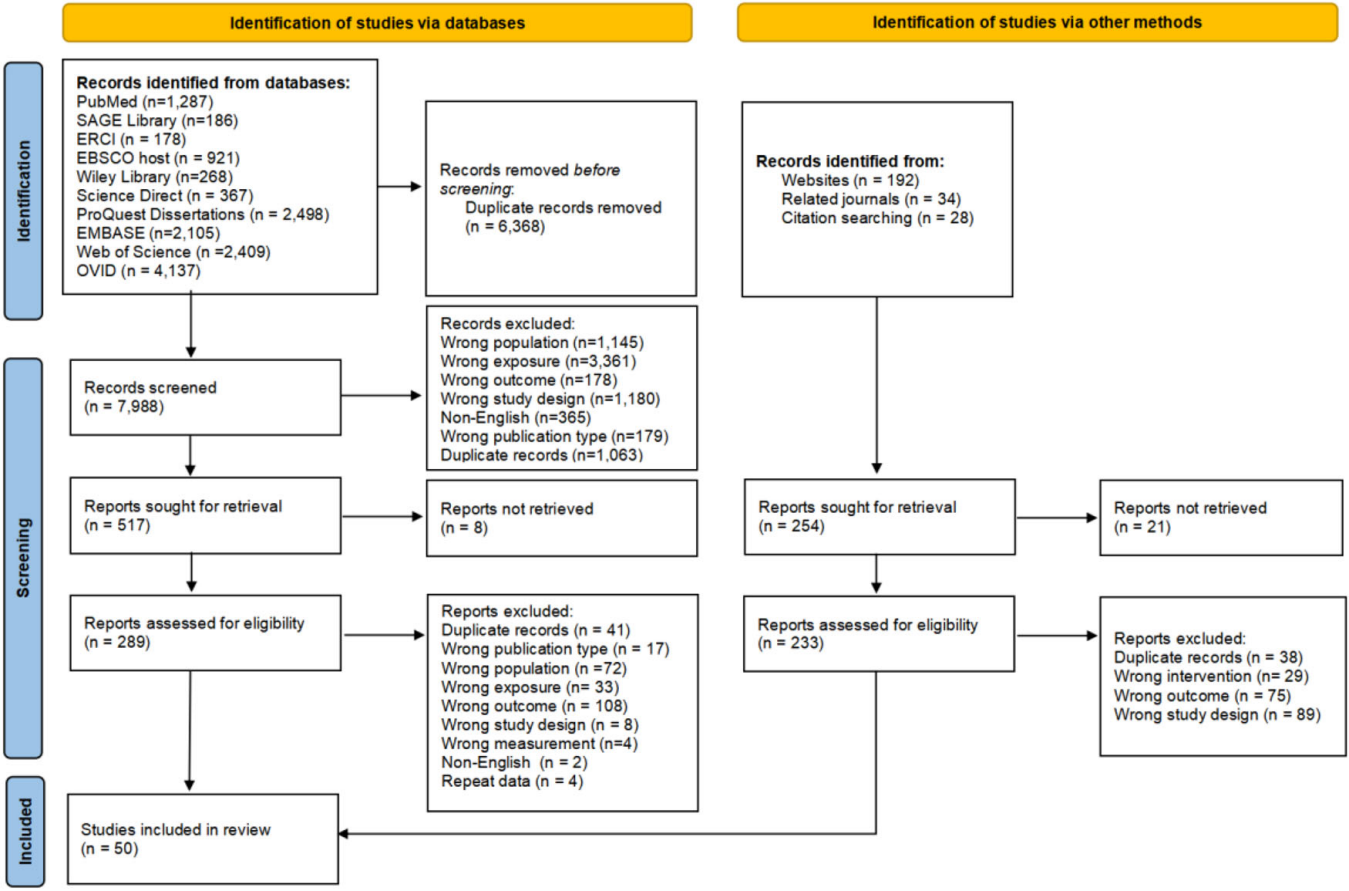
Flow diagram of literature search and processing of records.

Systematic retrieval of the electronic database was performed, including SAGE, ERIC, EBSCO, Wiley, ScienceDirect, ProQuest, PubMed, EMBASE, and Web of Science database. The detail of search strings was displayed in Supplementary (see Table S1). We set no restrictions on the year of publication. Additionally, the reference lists of eligible studies and relevant review articles, as well as the related meta‐analysis on the mental health of graduate students were searched manually. The deadline for searching is up to November 22, 2022. The process of the retrieval and selection of primary studies was achieved by the first two authors, and any inconsistency was solved by the third author. The process of the literature search and the selection of articles was based on the PRISMA flow diagram (see Figure 1).

### 3.2 Inclusion and exclusion criteria

To be included in the meta‐analysis, a primary study had to meet the following criteria: (a) the study population were healthy graduate students who aimed to pursue a master's or a doctoral degree, postgraduate‐training residents in medicine and other specializations were excluded; (b) the exposure was common anxiety, which measured by standardized instruments; the special anxiety such as social anxiety, death anxiety and performance anxiety were excluded; (c) the outcomes included the prevalence of anxiety symptom or can be referred from the paper; the studies missing total sample were excluded; (d) the studies used cross‐sectional design were included, experimental studies were excluded; (e) the article was published in English.

### 3.3 Information extraction and coding

Information extraction and coding included three parts: general information of study, the character of the sample and the outcomes. The general information of studies includes the title, author, the year of publication and collection; the character of the sample includes the information of sampling methods, sample size, the proportion of female, educational level, region; the outcomes includes methodological features (e.g., measurements, score system, score method, and standard for detection of anxiety) and effect size (prevalence of anxiety among graduate students). This progress conducted by the first two authors of the article and disagreements between coders were resolved by the third author.

### 3.4 Quality assessment

A modified version of the Newcastle‐Ottawa scale was used to assess the quality of studies included in meta‐analyses (Matcham et al., 2013). There are five domains: sampling method, sample size, participation rate, measurement tools, standards used to identify the anxiety disorder in the study (see Table S2 in supplement). Studies were rated as low quality (0–3 points), low to medium quality (4–6 points), medium to high quality (7–8 points), and high quality (9–10 points).

### 3.5 Data synthesis and analysis

Random‐effect model allowed to generalize findings from the included studies to a range of scenarios and larger population, a random‐effects meta‐analysis was performed in this review (Borenstein et al., 2009; Yaow et al., 2022). *Q* statistics and *I* ^2^ index were used to evaluate heterogeneity between studies. The *I* ^2^ index refers to the truly observed variation ratio (Borenstein et al., 2009), and 25%, 50%, and 75% of the *I* ^2^ respectively indicate low, medium, and high heterogeneity (Higgins & Thompson, 2002). Besides, “leave‐one‐out” method was adopted for sensitivity analysis to check for outliers that potentially influence the overall results (Cooper et al., 2009). Visual funnel plots and the “Fail‐safe N”method was conducted to evaluate potential publication bias. The fail‐safe N is the number of excluded studies with null results (i.e., zero effect sizes) that would be needed to bring their inclusion to lower the average effect size to a nonsignificant level (Rosenthal, 1979). Meta‐regression was used to identify the changes of anxiety prevalence, and stratified analysis in subgroups was used to explore the heterogeneity.

## 4 RESULTS

Fifty studies were included in the meta‐analysis. The data were collected from the United States (*n* = 19), the United Kingdom (*n* = 2), China (*n* = 15), India (*n* = 4), Australia (*n* = 1), Jordan (*n* = 1), Egypt (*n* = 1), Poland (*n* = 1), Croatia (*n* = 1), South Africa (*n* = 1), Nepal (*n* = 1), and Saudi Arabia (*n* = 1). The sample size ranged from 40 to 4477, totaling 39,668 graduate students. The proportion of females in each study ranged from 34.7% to 100%. The data were collected between 2005 and 2021, and 14 measures were used in primary studies. The details were shown in Table 1.

**TABLE 1:** Characters of primary studies included in the meta‐analysis

**Author, year**	**Year** ^a^	**Sampling method**	**Sample**	**Female (%)**	**Age *M* (SD)/Rang**	**Level**	**Country**	**Measure**	**Score method**	**Criteria of identification**	**QA**
Abu Ruz et al. (2018)	NR	Nonrandom	40	NR	NR	Both	Jordan	HADS	0–3	Mild: 8–10 Moderate: 11–14 Severe: 15–21	L‐M
Addonizio (2011)	2010	Nonrandom	367	90.74%	18–64	Master	US	K10	1–5	Mild: 20–24 Moderate: 25–29 Severe: 30 and over	M‐H
Ahuja and Kumar (2015)	2013	Nonrandom	40	100.00%	20–30	Both	NR	HAM‐A	NR	NR	L
Aleena et al. (2020)	NR	Nonrandom	400	50.00%	23+	Doctoral	China	DASS‐21	0–3	NR	M‐H
Allen et al. (2022)	2017	Nonrandom	2683	62.99%	28/20–60	Both	US	BAI	0–3	Mild: 8–15 Moderate: 16–25 Severe: 26 and over	M‐H
Almasri et al. (2022)	2020	Nonrandom	308	45.13%	NR	Doctoral	US	GAD‐7	NR	NR	L‐M
Al‐Shayea (2014)	2013	NR	79	53.16%	32/25–45	Both	Saudi Arabia	DASS‐21	0–3	Mild: 8–9 Moderate: 10–14 Severe: 15–19 Extremely severe: 20 and over	L‐M
Atkinson (2020)	2019	NR	84	100.00%	NR	Both	Australia	DASS‐21	0–3	Mild: 8–9 Moderate: 10–14 Severe: 15–19 Extremely severe: 20 and over	L‐M
Attia and Shata (2013)	2013	NR	155	73.55%	32.18(6.85)/ 22–52	Both	Egypt	DASS‐21	0–3	NR	L‐M
Balakrishnan et al. (2022)	2020	Nonrandom	90	78.89%	20–50	Both	US	DASS‐21	0–3	Mild: 8–9 Moderate: 10–14 Severe: 15–19 Extremely severe: 20 and over	L
Barreira et al. (2018)	2017–2018	Nonrandom	510	34.71%	20+	Doctoral	US	GAD‐7	0–3	Mild: 5–9 Moderate: 10–14 Severe: 15 and over	M‐H
Barton and Bulmer (2017)	2010–2017	Nonrandom	4477	59.37%	21+	Both	US	PHQ‐9	NR	NR	M‐H
Coakley et al. (2021)	2020	NR	530	NR	NR	Both	US	GAD‐7	0–3	Mild: 5–9 Moderate: 10–14 Severe: 15 and over	M‐H
Cullinan et al. (2022)	2018	Nonrandom	976	NR	NR	Both	UK	DASS‐21	0–3	Mild to moderate: 8–14 Severe to extremely severe: 15 and over	M‐H
Duan et al. (2022)	2020–2021	Nonrandom	342	NR	NR	Both	China	ZSAS	1–4	Mild: 50–59 Moderate: 60–69 Severe: 70 and over	M‐H
Eisenberg et al. (2007)	2005	Nonrandom	1662	49.28%	70% were 23–30	Both	US	PHQ‐9	NR	NR	L‐M
Ellison et al. (2020)	2018	NR	59	81.36%	25.48/23–36	Doctoral	US	DASS‐21	0–3	NR	L‐M
Evans et al. (2017)	NR	Nonrandom	2279	70.38%	NR	90% doctoral	26 countries	GAD‐7	NR	NR	L‐M
Fang et al. (2019)	2018	NR	3669	57.62%	22–28	Master	China	GAD‐7	NR	Positive symptom: 5 and over	L‐M
Garcia‐Williams et al. (2014)	2010 and 2012	Nonrandom	301	77.08%	27.98 (5.903)/18–63	Both	US	PHQ‐9	0–3	NR	L‐M
Ghogare et al. (2021)	2020	Nonrandom	195	71.79%	25–35	Both	India	DASS‐21	0–3	Mild: 8–9 Moderate: 10–14 Severe: 15–19 Extremely severe: 20 and over	L‐M
Hoying et al. (2020)	2017–2018	Nonrandom	197	NR	24.5 (4.9)	Both	US	GAD‐7	0–3	Mild: 5–9 Moderate: 10–14 Severe: 15 and over	L‐M
Jennifer et al. (2022)	2020	Nonrandom	1916	85.80%	20–45	Both	US	DASS‐21	NR	NR	L‐M
Jones‐White et al. (2022)	2017–2018	Random	2582	55.46%	18+	Doctoral	US	GAD‐2	0–3	Positive symptom: 3 and over	M‐H
Kowalczyk et al. (2021)	NR	NR	528	55.49%	26–30	Doctoral	Poland	GHQ‐28	0–3	Positive symptom: 28 and over	M‐H
Li et al. (2021)	2020	Random	339	82.60%	90% was 21–30	Both	China	SCL‐90	1–5	Positive symptom: subscale score over 2	H
Liang et al. (2021)	2020	Nonrandom	3137	78.29%	NR	Both	China	SAS	1–4	Mild: 50–59 Moderate: 60–69 Severe: over 69	M‐H
Liu et al. (2019)	2017	Nonrandom	325	60.31%	31.1 (5.3)/ 23–47	Doctoral	China	GAD‐7	0–3	Mild: 5–9 Moderate: 10–14 Severe: 15 and over	M‐H
Liu et al. (2020)	2017	NR	1625	54.15%	NR	Both	China	GAD‐7	0–3	Positive symptom: 10 and over	M‐H
Madhan et al. (2012)	2010	Nonrandom	330	44.85%	26 (1.8)/ 24–34	Master	India	DASS‐21	0–3	Mild: 8–9 Moderate: 10–14 Severe: 15–19 Extremely severe: 20 and over	M‐H
Mazurek Melnyk et al. (2016)	2014–2015	Nonrandom	91	65.93%	25.43/21–51	Both	US	GAD‐7	0–3	NR	L
Milicev et al. (2021)	2018–2019	NR	479	69.31%	31.1 (9.1)/21–73	Both	UK	GAD‐7	0–3	Mild: 5–9 Moderate: 10–14 Severe: 15 and over	M‐H
Negi et al. (2019)	NR	NR	76	50.00%	21–35	Both	India	DASS‐21	0–3	Mild: 8–9 Moderate: 10–14 Severe: 15–19 Extremely severe: 20 and over	L‐M
Peng et al. (2022)	2020–2021	Nonrandom	1410	73.48%	23–26	Both	China	GAD‐7	0–3	Positive symptom: 10 and over	M‐H
Pervez et al. (2021)	2019 and 2020	NR	113	49.56%	NR	Both	US	GAD‐7	0–3	Mild: 5–9 Moderate: 10–14 Severe: 15 and over	L‐M
Ramadoss and Horn (2022)	2021	NR	99	56.57%	NR	Doctoral	US	GAD‐2	0–3	Positive symptom: 3 and over	L‐M
Rosenthal et al. (2021)	2020	NR	222	NR	26–40	Both	US	DASS‐21	0–3	NR	L
Shadowen et al. (2019)	2017	Nonrandom	344	NR	NR	Both	US	BAI	0–3	Positive symptom: 19 and over	L‐M
Shete and Garkal (2015)	NR	NR	50	66.00%	23–34	Both	India	DASS‐42	0–3	Mild: 8–9 Moderate: 10–14 Severe: 15–19 Extremely severe: 20 and over	L
Talapko et al. (2021)	2021	Random	370	84.32%	NR	Both	Croatia	DASS‐21	0–3	NR	M‐H
Vuuren et al. (2021)	NR	NR	108	NR	NR	Both	South Africa	ZSAS	0–3	Mild to moderate: 45–59 Severe: 60–74 Extremely severe: 75 and over	L‐M
Wang et al. (2018)	2017	Random	260	44.62%	NR	Both	China	SCL‐90	1–5	Positive symptom: subscale score over 2	H
Wang et al. (2020)	2020	Nonrandom	375	NR	NR	Master	China	GAD‐7	0–3	Positive symptom: 5 and over	L‐M
Wu et al. (2022)	2021	NR	1336	52.40%	20+	Both	China	ZSAS	1–4	Positive symptom: 50 and over	M‐H
Xiao et al. (2020)	2020	NR	313	NR	NR	Both	China	GAD‐7	0–3	Mild: 5–9 Moderate: 10–14 Severe: 15 and over	M‐H
Yadav et al. (2021)	2020	Nonrandom	409	83.13%	22.10(2.928)	Both	Nepal	GAD‐7	0–3	Mild: 5–9 Moderate: 10–14 Severe: 15 and over	M‐H
Zakeri et al. (2021)	2020	Nonrandom	238	67.23%	NR	Doctoral	US	CCAPS‐62	0–4	Moderate: average sub scale‐score 1.22–1.89 Severe: average subscale score over 1.89	L‐M
Zhang et al. (2022)	2021	Random	911	78.59%	NR	Both	China	ZSAS	1–4	Mild: 50–59 Moderate: 60–69 Severe: 70 and over	H
Zhou et al. (2018)	2017	Nonrandom	1159	63.76%	NR	Both	China	SCL‐90	1–5	Positive symptom: Subscale score over 2	L‐M
Zhou et al. (2021)	2020	NR	1080	NR	NR	Both	China	GAD‐7	0–3	Mild: 5–9 Moderate: 10–14 Severe: 15 and over	M‐H

^a^ The end year of data collection.

NR: not report; QA: quality assessment; L: low quality (0–3 points); L‐M: low to medium quality (4–6 points); M‐H: medium to high quality (7–8 points); H: high quality (9–10 points); HADS: Hospital Anxiety and Depression Scale; K10: Kessler Psychological Distress Scale; HAM‐A: Hamilton Anxiety Scale; BAI: Beck Anxiety Inventory; DASS: Depression Anxiety and Stress Scale; GAD‐7: Generalized Anxiety Disorder 7‐item; PHQ: Patient Health Questionnaire; SAS: Self‐Rating Anxiety Scale; SCL‐90: Symptom Check List 90; CCAPS‐62: Counseling Center Assessment of Psychological Symptoms; ZSAS: Zung's Self‐Rating Anxiety Scale; GHQ‐28: General Health Questionnaires.

### 4.1 Quality assessment

Quality assessment of primary studies including five domains: sampling method, sample size, participation rate, measurement method, and criteria for identifying the anxiety disorder. Five of 50 (10.0%) reports were in high quality, 21 (42%) were in moderate to high quality, 21 (42%) were in low to moderate quality, and 3 (6%) were in low quality (Table 1). We conducted a subgroup analysis based on the quality of included studies; the results showed that studies assessed with low quality reported higher prevalence of anxiety than that with high quality (any anxiety: 40.3% vs. 13.0%, *Q* = 11.7, *df* = 4, *p* = .01), and studies assessed with low to moderate quality reported higher prevalence of moderate and severe anxiety than that with high quality (moderate: 24.7% vs. 4.7%, *Q* = 59.8, *df* = 3, *p <* .00; severe: 15.1% vs. 1.4%, *Q* = 54.5, *df* = 3, *p* < .00).

### 4.2 Sensitivity analysis

With the method of leave‐one‐out method, we got 50 effect sizes of any anxiety, ranging from 32.8% to 36.0% (*p* < .00), 23 of mild anxiety, ranging from 18.8% to 20.2% (*p* < .00), 24 of moderate anxiety, ranging from 4.7% to 73.7% (*p* < .00), and 25 of severe anxiety, ranging from 0.9 % to 32.5% (*p* < .00). One study (Negi et al., 2019) was biased against the results of moderate anxiety, which was removed in the later analysis.

### 4.3 Publication bias

**FIGURE 2:**
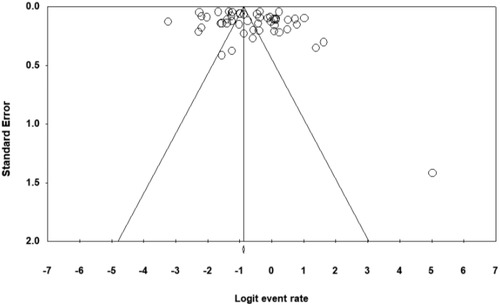
Funnel plot of anxiety prevalence among graduate students.

The funnel plots suggested that the studies were symmetrically distributed (Figure 2), and the fail‐safe number was larger than the recommended criteria (5*K* + 10, *K* means the number of studies included in meta‐analysis) in the prevalence of any anxiety (*N_fs_* = 37,109 > 5*K* + 10 = 260), mild anxiety (*N_fs_* = 13,409 > 5*K* + 10 = 125), moderate anxiety (*N_fs_* = 14,744 > 5*K* + 10 = 125), and severe anxiety (*N_fs_* = 16,514 > 5*K* + 10 = 135). These results showed that there was no potential publication bias.

### 4.4 Prevalence of anxiety among graduate students

Fifty studies involved the any anxiety among graduate students with the prevalence ranging from 3.8% to 100.0%. Meta‐analytic pooling of the prevalence estimates of anxiety was 34.8% (*N* = 39,668; 95% CI: 29.5%−40.5%), with significant heterogeneity across studies (*Q* = 4683.1, *p* < .00; *I* ^2^= 98.9%). As shown in Table 2, 19.1% of them suffered mild anxiety (*K* = 23, *N* = 11,426; 95% CI: 15.4%−23.5%; *Q* = 513.3, *p* < .00; *I* ^2^= 95.7%), 15.1% moderate anxiety (*K* = 23, *N* = 10,720; 95% CI: 11.6%−19.6%;*Q* = 539.7, *p* < .00; *I* ^2^= 95.9%), and 10.3% severe anxiety (*K* = 25, *N* = 1850; 95% CI: 7.2%–14.6%; *Q* = 889.7, *p* < .00; *I* ^2^= 97.3%).

**TABLE 2:** The results of subgroup analysis of anxiety prevalence among graduate students

	**Any anxiety**					**Mild anxiety**					**Moderate anxiety**					**Severe anxiety**				
	*K*	*E*s	LL	UL	*I* ^2^ (%)	*K*	*E*s	LL	UL	*I* ^2^ (%)	*K*	*E*s	LL	UL	*I* ^2^ (%)	*K*	*E*s	LL	UL	*I* ^2^ (%)
***Quality of studies***			***Q* = 11.704** ^**^		**98.95**			***Q* = 6.059** ^**^		**95.71**			***Q* = 69.674** ^**^		**95.92**			***Q* = 54.480** ^**^		**97.30**
L	5	0.403	0.233	0.600	92.88	3	0.093	0.014	0.419	94.22	3	0.176	0.083	0.335	83.00	3	0.093	0.060	0.141	13.09
L‐H	21	0.391	0.293	0.500	98.96	9	0.166	0.096	0.270	95.47	10	0.214	0.173	0.262	82.14	11	0.151	0.107	0.209	9.71
M‐H	21	0.341	0.268	0.423	99.19	10	0.226	0.176	0.286	96.31	9	0.113	0.069	0.180	97.34	10	0.087	0.045	0.160	98.36
H	3	0.130	0.066	0.241	94.60	1	0.159	0.137	0.184	*/*	1	0.047	0.035	0.063	*/*	1	0.014	0.008	0.024	*/*
***Sampling method***			***Q* = 3.334**		**99.10**			***Q* = 3.087**		**96.43**			***Q* = 6.877** ^**^		**97.10**			***Q* = 0.004**		**98.36**
Nonrandom	27	0.336	0.264	0.416	99.17	12	0.206	0.155	0.269	96.90	12	0.133	0.090	0.192	97.10	13	0.083	0.047	0.143	98.34
Random	5	0.207	0.123	0.327	98.15	2	0.156	0.137	0.177	0.00	2	0.059	0.037	0.094	75.53	2	0.075	0.003	0.698	99.22
***Measure***		***Q* = 528.159** ^**^			**98.95**		***Q* = 253.005** ^**^			**95.71**		***Q* = 207.174** ^**^			**95.92**		***Q* = 256.617** ^**^			**97.30**
BAI	2	0.200	0.149	0.262	82.72	0	/	/	/	/	0	/	/	/	/	0	/	/	/	/
CCAPS‐62	1	0.496	0.433	0.559	/	0	/	/	/	/	1	0.252	0.201	0.311	.00	1	0.244	0.193	0.302	/
DASS‐21	13	0.558	0.483	0.631	95.06	12	0.147	0.103	0.206	95.00	10	0.194	0.155	0.241	87.64	12	0.184	0.142	0.236	93.39
DASS‐42	1	0.800	0.667	0.889	/	1	0.360	0.240	0.501	.00	1	0.320	0.206	0.460	.00	1	0.120	0.055	0.242	/
GAD‐2	2	0.305	0.163	0.496	93.53	0	/	/	/	/	0	/	/	/	/	0	/	/	/	/
GAD‐7	16	0.353	0.269	0.448	98.93	6	0.318	0.298	0.339	1.83	6	0.138	0.102	0.184	85.75	6	0.077	0.041	0.138	93.41
GHQ‐28	1	0.199	0.167	0.235	/	0	/	/	/	/	0	/	/	/	/	0	/	/	/	/
HADS	1	0.225	0.121	0.379	/	0	/	/	/	/	0	/	/	/	/	0	/	/	/	/
HAM‐A	1	0.175	0.086	0.324	/	1	0.025	0.004	0.157	/	1	0.130	0.056	0.273	/	1	0.025	0.004	0.157	/
K10	1	0.518	0.467	0.568	/	0	/	/	/	/	0	/	/	/	/	0	/	/	/	/
PHQ‐9	3	0.142	0.032	0.454	99.55	0	/	/	/	/	0	/	/	/	/	0	/	/	/	/
SAS	1	0.209	0.195	0.224	/	1	0.147	0.135	0.160	/	1	0.047	0.040	0.054		1	0.016	0.012	0.021	/
SCL‐90	3	0.111	0.097	0.126	/	0	/	/	/	/	0	/	/	/	/	0	/	/	/	/
ZSAS	4	0.248	0.197	0.307	88.33	2	0.153	0.135	0.175	.00	3	0.078	0.018	0.282	97.36	3	0.022	0.006	0.075	88.21
***Education level***			***Q* = 0.36**		**98.67**			***Q* =0 .038**		**88.31**			***Q* = 6.855** ^**^		**86.88**			***Q* =0 .053**		**95.35**
Master	9	0.292	0.180	0.437	99.01	2	0.237	0.212	0.263	.00	1	0.261	0.216	0.311	/	6	0.135	0.068	0.252	47.71
Doctoral	14	0.343	0.252	0.447	98.25	5	0.229	0.160	0.315	91.75	5	0.165	0.121	0.220	83.82	6	0.135	0.068	0.252	96.47
***Gender***			***Q* = 0.374**		**98.63**			***Q* = 0.031**		**86.07**			***Q* = 0.029**		**89.68**			***Q* = 0.131**		**91.78**
female	16	0.412	0.291	0.544	98.88	7	0.223	0.145	0.328	85.13	8	0.241	0.162	0.344	88.26	8	0.150	0.092	0.235	86.95
male	14	0.355	0.240	0.490	98.34	5	0.210	0.118	0.346	88.94	6	0.256	0.142	0.416	91.98	6	0.123	0.043	0.303	95.12
***Country***			***Q* = 237.678** ^**^		**98.96**			***Q* = 6.069** ^**^		**95.87**			***Q* = 102.735** ^**^		**96.10**			***Q* = 98.458** ^**^		**97.40**
Australia	1	0.560	0.452	0.662	/	1	0.131	0.074	0.221	/	1	0.250	0.169	0.353	/	1	0.178	0.110	0.275	/
China	15	0.220	0.170	0.281	98.48	5	0.177	0.129	0.238	94.60	5	0.073	0.035	0.146	97.39	5	0.037	0.007	0.184	99.10
Croatia	1	0.532	0.481	0.583	/	1	0.149	0.116	0.189	/	1	0.076	0.053	0.108	/	1	0.307	0.262	0.356	/
Egypt	1	0.523	0.444	0.600	/	1	0.103	0.064	0.162	/	1	0.213	0.156	0.284	/	1	0.207	0.150	0.278	/
India	4	0.727	0.606	0.821	81.44	4	0.252	0.164	0.365	81.47	3	0.274	0.239	0.312	.00	4	0.141	0.092	0.209	72.35
Jordan	1	0.225	0.121	0.379	/	0	/	/	/	/	0	/	/	/	/	0	/	/	/	/
Nepal	1	0.472	0.424	0.520	/	1	0.315	0.272	0.362	/	1	0.103	0.077	0.136	/	1	0.054	0.036	0.081	/
Poland	1	0.199	0.167	0.235	/	0	/	/	/	/	0	/	/	/	/	0	/	/	/	/
Saudi Arabia	1	0.835	0.737	0.902	/	1	0.367	0.269	0.478	/	1	0.342	0.246	0.453	/	1	0.126	0.069	0.219	/
South Africa	1	0.361	0.276	0.456	/	0	/	/	/	/	1	0.287	0.210	0.379	/	1	0.074	0.037	0.141	/
UK	2	0.396	0.371	0.421	.00	1	0.240	0.214	0.268	/	0	/	/	/	/	1	0.151	0.130	0.174	/
US	19	0.351	0.252	0.466	99.29	7	0.181	0.099	0.306	97.92	8	0.161	0.130	0.198	82.99	8	0.128	0.079	0.201	96.04
***COVID‐19***			***Q* = 0.452**		**98.96**			***Q* = 1.210**		**95.86**			***Q* = 1.683**		**96.10**			***Q* = 1.033**		**97.34**
before	20	0.362	0.287	0.443	99.02	12	0.224	0.180	0.275	85.46	9	0.177	0.127	0.241	87.59	12	0.119	0.087	0.159	84.85
after	27	0.324	0.253	0.404	98.68	10	0.180	0.129	0.246	97.27	12	0.125	0.082	0.187	97.41	11	0.082	0.041	0.155	98.58

*p* < .05.

** *p* < .01.

### 4.5 The trend of anxiety prevalence among graduate students

**FIGURE 3:**
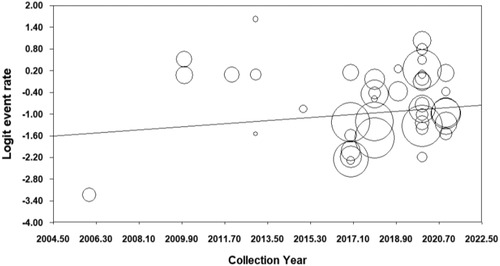
The prevalence of any anxiety among graduates through the year.

The data we included were collected between 2005 and 2021, crossed 17 years. The result of fixed‐effect meta‐regression analysis showed that the prevalence of anxiety increased slightly among postgraduates (*β* = .05, *p* < .00;Figure 3), especially in severe anxiety (*β* = .07, *p* < .00), but the prevalence of mild and moderate anxiety decreased significantly through the year (mild anxiety: *β* = –.07, *p <* .00; moderate anxiety: *β* = –.09, *p* < .00).

To explore the effect of COVID‐19, we compared the prevalence of anxiety between before pandemic (before 202) and after (2020–2021) based on the data collection year. The results showed that there was no significant difference of the anxiety prevalence (any anxiety: 32.4% vs. 36.2%; *Q* = 0.45, *df* = 1, *p* = .50; mild anxiety: 22.4% vs. 18.0%; *Q* = 1.21, *df* = 1, *p* = .27; moderate anxiety: 21.5% vs. 12.5%; *Q* = 3.19, *df* = 1, *p* = .07; severe anxiety: 11.9% vs. 8.2%; *Q* = 1.03, *df* = 1, *p* = .31).

### 4.6 Prevalence of anxiety mediated by moderators

#### 4.6.1 Study‐level characteristics

***Sampling method***. Five studies were collecting data with random sampling, 27 studies adopted convenience sampling method, and 18 studies did not report the sampling method. The results of subgroup analysis showed that the studies with nonrandom sampling reported a higher prevalence of moderate anxiety than that with random sampling (any anxiety: 33.6% vs. 2.7%; *Q* = 3.33, *df* = 1, *p* = .07; mild: 2.6% vs. 15.6%; *Q* = 3.09, *df* = 1, *p* = .08; moderate: 13.3% vs. 5.9%; *Q* = 6.88, *df* = 1, *p* = .01; severe: 8.3% vs. 7.5%; *Q* = 0.01, *df* = 1, *p* = .95).

***Measures***. Although we only included the studies using standardized instruments, the criteria for anxiety were various, which is a potential source of heterogeneity. The result of subgroup analysis showed indicated that DASS‐42 had a higher sensitivity of anxiety, especially for mild and moderate anxiety (any anxiety: 8.0%, *Q* = 528.2, *df* = 13, *p <* .00; mild: 36.0%, *Q* = 253.0, *df* = 5, *p <* .00; moderate: 32.0%, *Q* = 203.0, *df* = 6, *p <* .00); DASS‐21 had a higher sensitivity of severe anxiety than other measures (18.4%, *Q* = 256.6, *df* = 6, *p <* .00). While the results using GAD‐7 is closer to the results of meta‐analysis (any anxiety: 35.3% vs. 34.8%; mild: 31.8% vs. 19.1%; moderate: 13.8% vs. 15.1%; severe: 7.7% vs. 1.3%).

***Gender***. Sixteen studies compared the anxiety prevalence between female and male; the results of subgroup analysis showed that there was no evidence indicated the significant difference between them (any anxiety: 41.2% vs. 35.5%; *Q* = 0.37, *p* = .54; mild anxiety: 22.3% vs. 21.0%; *Q* = 0.03, *p* = .86; moderate anxiety: 24.1% vs. 25.6%; *Q* = 0.03, *p* = .86; severe anxiety: 15.0% vs. 12.3%; *Q* = 0.13, *p* = .72).

***Education level***. Nine studies reported the anxiety prevalence of master students, and 14 reported doctoral students. The results of subgroup analysis showed that master student suffered similar anxiety to doctoral students, and even higher moderate anxiety (any anxiety: 29.2% vs. 34.2%, *Q* = 0.36, *p* = .55; mild anxiety: 23.7% vs. 22.9%, *Q* = 0.04, *p* = .85; moderate anxiety: 26% vs. 16.5%, *Q* = 6.86, *p* = .01; severe: 14.7% vs. 13.5%, *Q* = 0.05, *p* = .82).

***Country***. The graduate students included in this meta‐analysis covered 12 countries, and the results of subgroup analysis showed that students from Saudi Arabia and India experienced most anxiety and students from China and the United States reported lowest anxiety than that from other countries. The details were shown in Table 2.

## 5 DISCUSSION

The present meta‐analysis estimated the population prevalence of anxiety disorder among graduate students. And the results indicated that 34.1% of them suffered from different degrees of anxiety. Of these samples included, 19.1% had mild anxiety, 15.1% moderate anxiety, and 1.3% had severe anxiety, which is significantly higher than college students (Ramón‐Arbués et al., 2020) and even higher than the medical student population (Quek et al., 2019). These results are consistent with the previous studies (Evans et al., 2017; Hoying et al., 2020), which revealed the high risk of anxiety disorder among postgraduate students. The factors of anxiety among graduate students are various and universal, such as employment prospects, inability to maintain work–life balance, inability to complete academic tasks on time, high academic competition, and long study period (Woolston, 2019).

Since 2005, countries around the world have recognized the seriousness of anxiety among graduate students and taken relevant measures (Lancet, 2015; Liu & Page, 2016; Mirza & Rahman, 2019), and the prevalence of mild and moderate anxiety decreased slightly. But the evidence suggested an increasing trend in any and severe anxiety; thus, we still need to keep going to prevent this from getting worse. Since the end of 2019, when COVID‐19 appeared, researchers indicated a significant increase of anxiety among among adults (Coley & Baum, 2021), but we found no obviously changing between before and after pandemic. It is mainly owing to the timely measures taken by school (Dempsey et al., 2022).

As the evidence quality, 84% of studies were in medium quality (including L‐M and M‐H), and the studies with low quality tended to reported a higher prevalence of anxiety, and studies with low to moderate quality reported higher prevalence of moderate and severe anxiety than that with high quality. All the studies (24, 48%) assessed into low quality or low to moderate quality adopted convenient sampling, and only two response rates in these studies were over 75% (Al‐Shayea, 2014; Negi et al., 2019). These factors may lead to an overestimate of anxiety prevalence; thus, it is essential to conduct a series high quality of primary in the following research.

We explored heterogeneity with study‐level characteristics and found that DASS‐42 had a higher sensitivity for anxiety, but compared to the results from the present meta‐analysis, the studies using GAD‐7 reported a representative result. Moreover, studies using nonrandom sampling reported an obvious higher prevalence of moderate anxiety than that using random sampling (13.3% vs. 5.9%). Convenience sample is drawn from a source that is conveniently accessible to researchers, but may not be representative of the population at large (Andrade, 2020). Majority (71.43%) studies included in the meta‐analysis were adopted convenience sampling, which may overestimate the prevalence of global graduate students. Thus, it is essential to conduct a large random sample survey from worldwide students in the further research.

Of note, we found that master students reported a similar prevalence of anxiety to doctoral students (29.2% vs. 34.3%), and even a higher prevalence of moderate anxiety (26% vs. 16.5%). Doctoral students always suffer with high pressure and stress; they have to keep a balance between life, work, and study in addition to academic pressure and employment pressure. According to the data from the Organization for Economic Co‐operation and Development (OCED) showed that the average share of younger adults with a tertiary degree has increased from 27% in 2000 to 48% in 2021 among 25‐ to 34‐years‐old (OCED., 2022), and the number of degree holders worldwide will reach 300 million by 2030 (OCED., 2013). This change of talent pool brings lots of pressure to master students, and they may lose their advantage from academic qualification in the job marketing in the future, which pushes them to pursue higher degree.

Besides, we found no obvious change of anxiety prevalence by gender; this was consistent with the previous studies on the mental health of graduate students. As the report from WHO indicated that more women are affected by mental illness than men (WHO, 2017), and based on the survey to 2279 graduate students across 26 countries, Evans et al. (2018) also presented that female suffered more major anxiety than male (43% vs. 34%) (Evans et al., 2018). But the results in the present review found no significant difference. One reason for this is that the source of anxiety in the graduate student population was similar.

In addition, students from Saudi Arabia and India experienced more anxiety than that from China and the United States. One reason is that 33 (66%) of the primary studies included were from China and the United States with 33,169 (83.6%) graduate students, resulting in unrepresentative data of other countries. Cross‐sectional studies with large samples from Saudi Arabia and India are required in the further research.

## 6 LIMITATIONS

The results of the present study showed that 34.8% of graduates suffered from anxiety, which indicated they were at the risk of mental health. While several limitations remained to consider these findings cautiously. First, although we considered different degree of anxiety symptom (e.g., mild, moderate and severe level), majority of participants were assessed through self‐report inventories rather than gold‐standard diagnostic clinical interviews for anxiety, which may lead to overestimate or underestimate the result. Meanwhile, we found a significant heterogeneity due to the different criteria for anxiety across these self‐rating scales. Second, majority of included studies adopted nonrandom sampling; as mentioned above, this may overestimate the prevalence of moderate anxiety. Lastly, the sample size from 36% studies was under 300, which means these data are not representative. Therefore, high‐quality studies with large sample are needed in the further research.

## 7 CONCLUSIONS

More than one‐third of graduate students are at the risk of anxiety distress, and this prevalence had a slightly upward since 2005. Although there was no obvious change after the COVID‐19 pandemic occurred, graduate schools should provide efficient program, such as psychological hotlines, mental health education, to prevent mental disorder of graduates for deteriorating. Teachers also need to improve their ability to identify the potential vulnerable students and take actions before their mental distress worse. Students are advised to seek the processional help when they feel depressed or anxious.

## AUTHOR CONTRIBUTIONS

TC and ZJZ conceived and drafted the study. TC and LYC analyzed, interpreted the data, drafted the manuscript, processed, and beautified the pictures and tables. ZJZ critically revised the manuscript. Ting Chi and Luying Cheng equally contributed to the work. All authors have approved the final draft of the manuscript.

## ROLE OF FUNDING

The funders had no role in the design and conduct of the study; collection, management, analysis, and interpretation of the data; preparation, review, or approval of the manuscript; and decision to submit the manuscript for publication.

## CONFLICT OF INTEREST STATEMENT

The authors declare they have no conflicts of interest.

### PEER REVIEW

The peer review history for this article is available at https://publons.com/publon/10.1002/brb3.2909.

## INFORMED CONSENT STATEMENT

This project is considered a service evaluation not directly influencing patient care or safety, and therefore, ethics approval was not required. Consent will be requested from all study participants. The final result will be published and freely available.

## Supporting information

Table S1. Search strategy and results (retrieval date: November 22, 2022).
Table S2. Items for quality assessment.

## Data Availability

The data presented in this study are available on request from the corresponding author.
