# Supplementary material for: Global prevalence and trend of anxiety among graduate students: A systematic review and meta‐analysis
Source: Brain Behav. 2023 Feb 27;13(4):e2909. doi: 10.1002/brb3.2909 (PMC10097092; doi:10.1002/brb3.2909)
Supplement: Supplementary file 1 — Table S1. Search strategy and results (retrieval date: November 22, 2022). Table S2. Items for quality assessment. [file BRB3-13-e2909-s001.docx]

**Table S1. Search strategy and results (retrieval date: 22 November 2022)**

| **Database** | **Search strategy** | **Record** |
| --- | --- | --- |
| PubMed | ("self rating anxiety scale sas"[Title/Abstract] OR "generalized anxiety disorder 7 item scale"[Title/Abstract] OR "gad"[Title/Abstract] OR "Anxiety"[Title/Abstract] OR "Anxiety"[MeSH Terms]) AND ("graduate*"[Title/Abstract] OR "postgraduate*"[Title/Abstract] OR "graduate student*"[Title/Abstract] OR "postgraduate student*"[Title/Abstract] OR "master student*"[Title/Abstract] OR "doctoral student*"[Title/Abstract] OR "doctor candidate"[Title/Abstract] OR "ph.d"[Title/Abstract]) | 1,287 |
| SAGE | ‘"self rating anxiety scale sas" OR "generalized anxiety disorder 7 item scale" OR GAD OR anxiety’ in abstract AND ‘graduate* OR postgraduate* OR "graduate student*" OR "master student*" OR "postgraduate student*" OR "doctoral student*" OR "doctor candidate" OR ph.d’ in abstract | 186 |
| ERIC | abstract: ( "self rating anxiety scale sas" OR "generalized anxiety disorder 7 item scale" OR GAD OR anxiety’ ) AND abstract: ( graduate* OR postgraduate* OR "graduate student*" OR "master student*" OR "postgraduate student*" OR "doctoral student*" OR "doctor candidate" OR ph.d ) | 178 |
| EBSCO | SU ( "self rating anxiety scale sas" OR "generalized anxiety disorder 7 item scale" OR GAD OR anxiety) AND SU ( graduate* OR postgraduate* OR "graduate student*" OR "master student*" OR "postgraduate student*" OR "doctoral student*" OR "doctor candidate" OR ph.d ) | 921 |
| Wiley | ""self rating anxiety scale sas" OR "generalized anxiety disorder 7 item scale" OR GAD OR anxiety’" in Abstract and "graduate* OR postgraduate* OR "graduate student*" OR "master student*" OR "postgraduate student*" OR "doctoral student*" OR "doctor candidate" OR ph.d" in Abstract | 268 |
| ScienceDirect | Title, abstract, keywords: anxiety AND ("graduate OR postgraduate OR "master student" OR "doctoral student") | 367 |
| ProQuest | abstract("self rating anxiety scale sas" OR "generalized anxiety disorder 7 item scale" OR GAD OR anxiety) AND abstract(graduate* OR postgraduate* OR "graduate student*" OR "master student*" OR "postgraduate student*" OR "doctoral student*" OR "doctor candidate" OR ph.d) | 2,498 |
| EMBASE | ('self rating anxiety scale sas':ti,ab,kw OR 'generalized anxiety disorder 7 item scale':ti,ab,kw OR gad:ti,ab,kw OR anxiety:ti,ab,kw) AND (graduate*:ti,ab,kw OR postgraduate*:ti,ab,kw OR 'graduate student*':ti,ab,kw OR 'master student*':ti,ab,kw OR 'postgraduate student*':ti,ab,kw OR 'doctoral student*':ti,ab,kw OR 'doctor candidate':ti,ab,kw OR ph.d:ti,ab,kw) | 2,105 |
| Web of Science | **#1** (((TS=("self rating anxiety scale sas")) OR TS=("generalized anxiety disorder 7 item scale")) OR TS=("GAD")) OR TS=(Anxiety)  **#2** (((((((TS=(graduate*)) OR TS=(postgraduate*)) OR TS=("graduate student*")) OR TS=("postgraduate student*")) OR TS=("master student*")) OR TS=("doctoral student*")) OR TS=("doctor candidate")) OR TS=(ph.d)  **#3** #1 AND #2 | 2,409 |
| OVID | (("self rating anxiety scale sas" or "generalized anxiety disorder 7 item scale" or GAD or anxiety) and (graduate* or postgraduate* or "graduate student*" or "master student*" or "postgraduate student*" or "doctoral student*" or "doctor candidate" or ph d)).ab. | 4,137 |
| **Total** |  | **14,356** |

**Table S2 Items for quality assessment**

| Items | Score |
| --- | --- |
| ***Representativeness*** |  |
| 1. What kind of recruitment strategy has been used? | Randomised/consecutive (2)  Non-randomised/convenience (0)  Not stated (0) |
| ***Sample Size*** |  |
| 1. What is the sample size? | 50-149 (0)  150-399 (1)  400+ (2) |
| ***Participation rate*** |  |
| 1. Is the participation rate reported? | Yes (2)  No (0) |
| 1. Is participation rate >75% | Yes (1)  No (0) |
| ***Criteria for anxiet***y |  |
| 1. How has anxiety been detected? | Screening tool (2)  Self-report questionnaire (0) |
| ***Eligibility Criteria*** |  |
| 1. Have eligibility criteria been specified? | Yes (1)  No (0) |
